# Supplementary material for: Melatonin and Coenzyme Q10 mitigate Senescence in Human Adipose-Derived Mesenchymal Stem Cells by Restoring Mitophagy and Mitochondrial Proteostasis
Source: PLoS One. 2026 Apr 29;21(4):e0347781. doi: 10.1371/journal.pone.0347781 (PMC13128124; doi:10.1371/journal.pone.0347781)
Supplement: S1 Fig — (PDF) [file pone.0347781.s001.pdf]

Parkin – 50kDa (Chemi Doc)

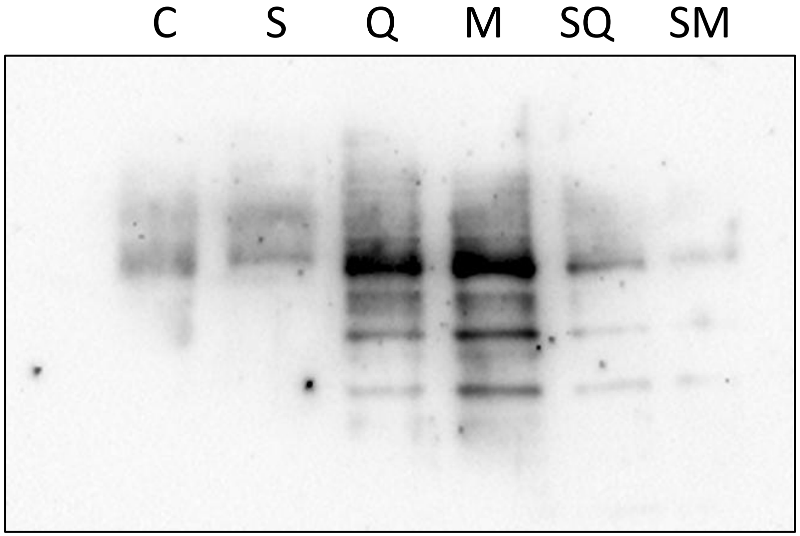

β-Actin – 42kDa (Chemi Doc)

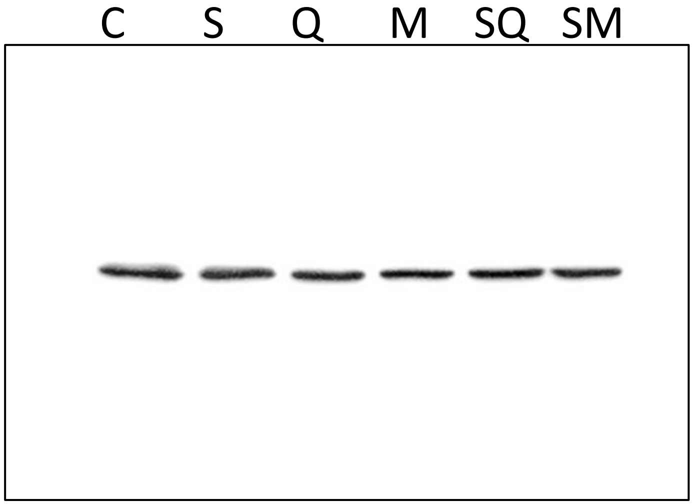

Parkin – 50kDa (merged)

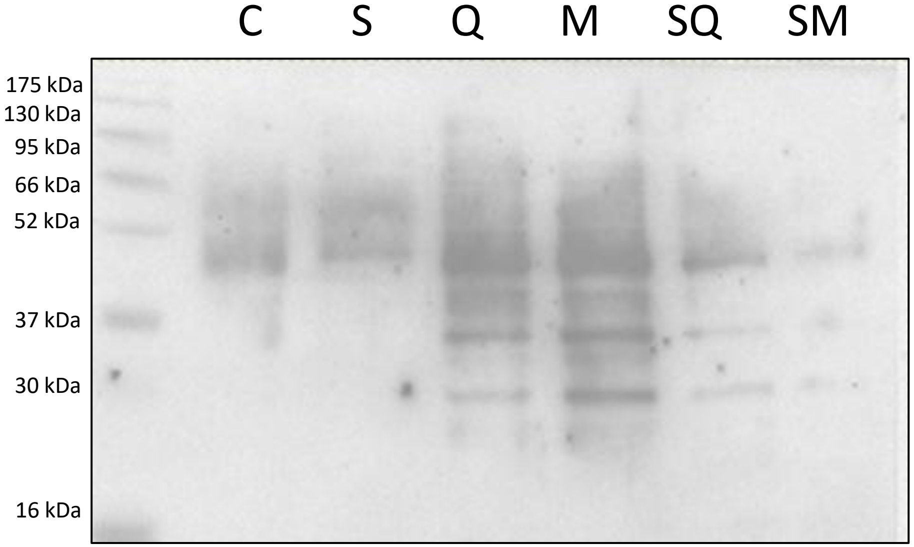

β-Actin – 42kDa (merged)

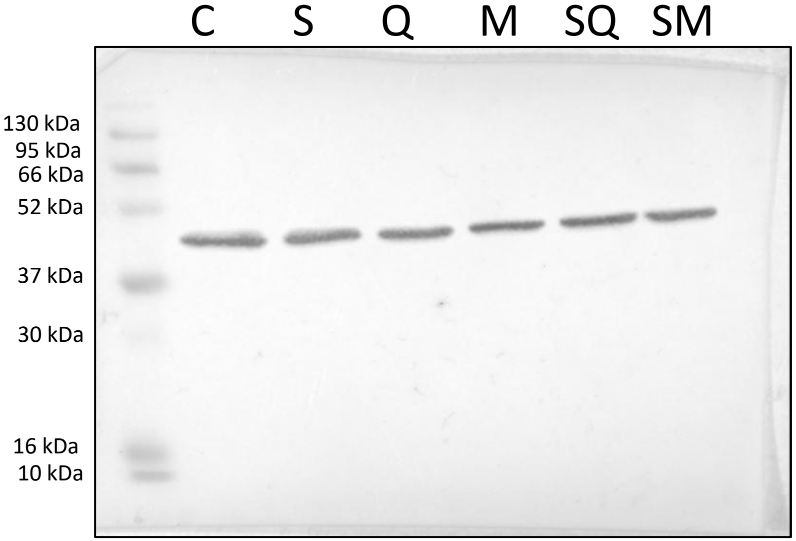

Optineurin 75kDa (Chemi Doc)

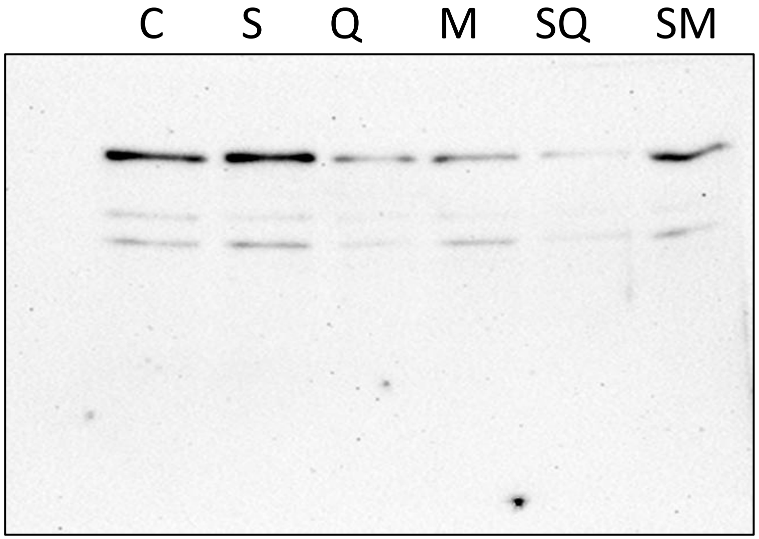

β -Actin -42kDa (Chemi Doc)

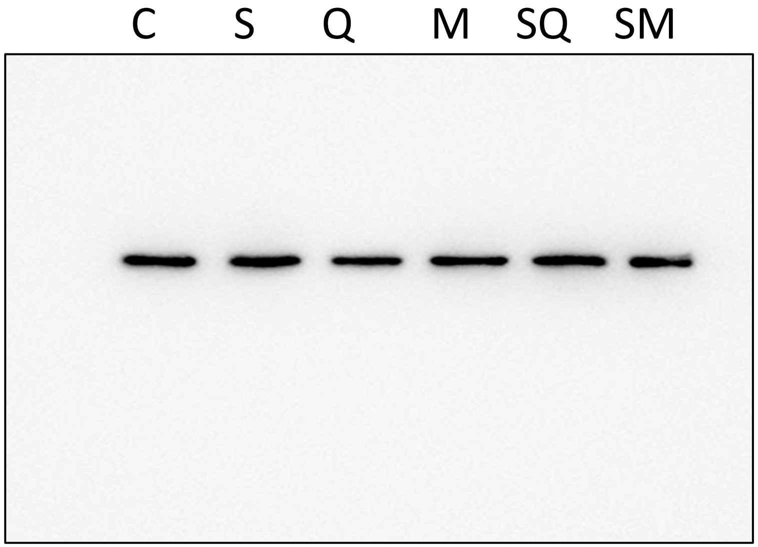

Optineurin 75kDa (merged)

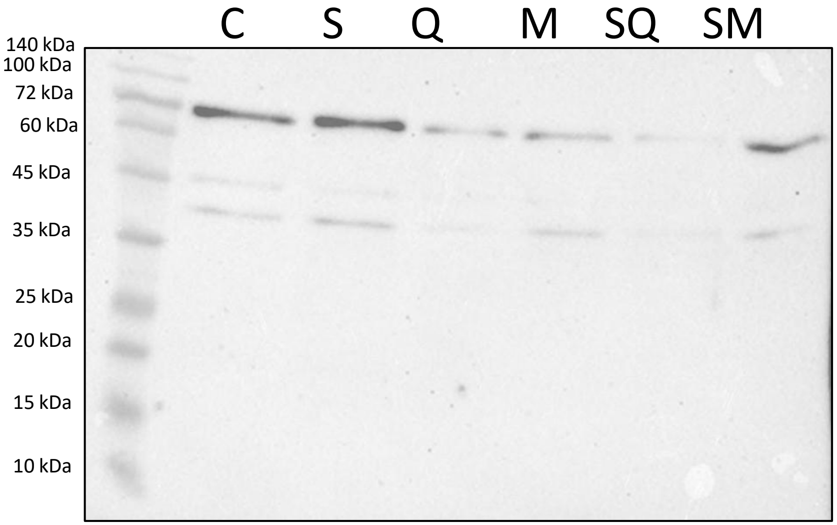

β -Actin -42kDa (merged)

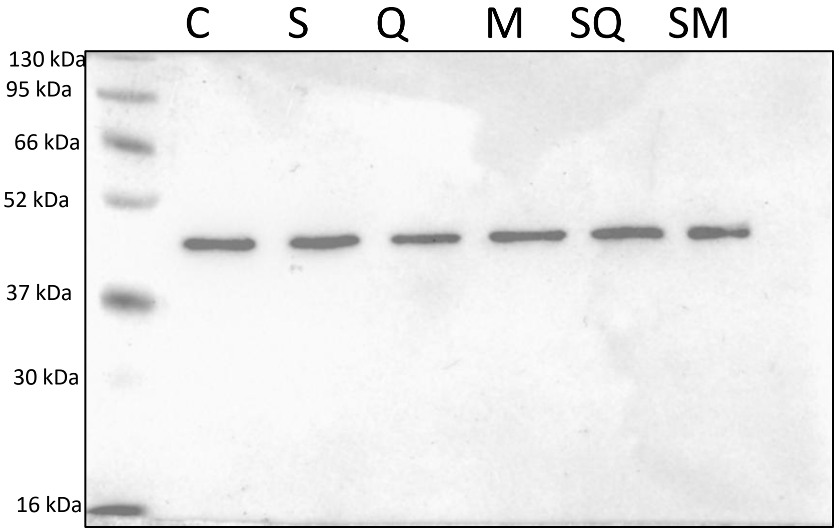

NDP52 – 52 kDa (Chemi Doc)

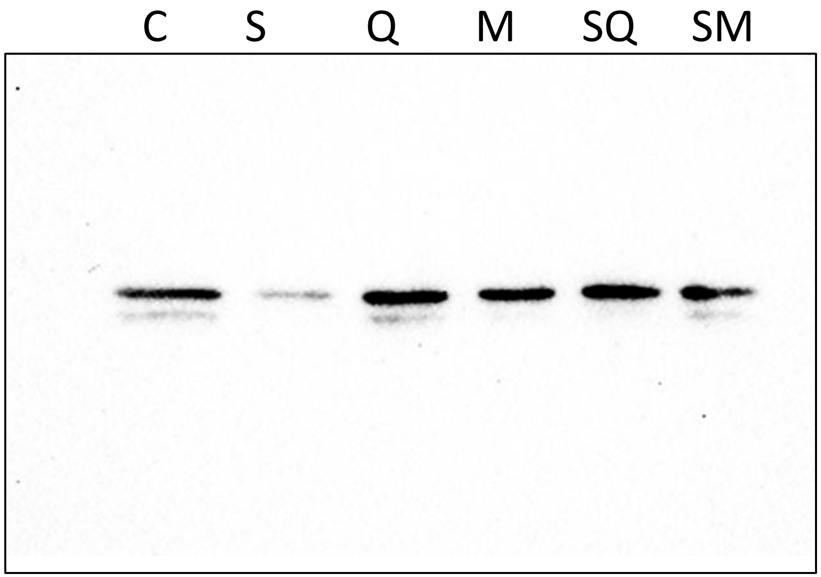

β -Actin -42kDa (Chemi Doc)

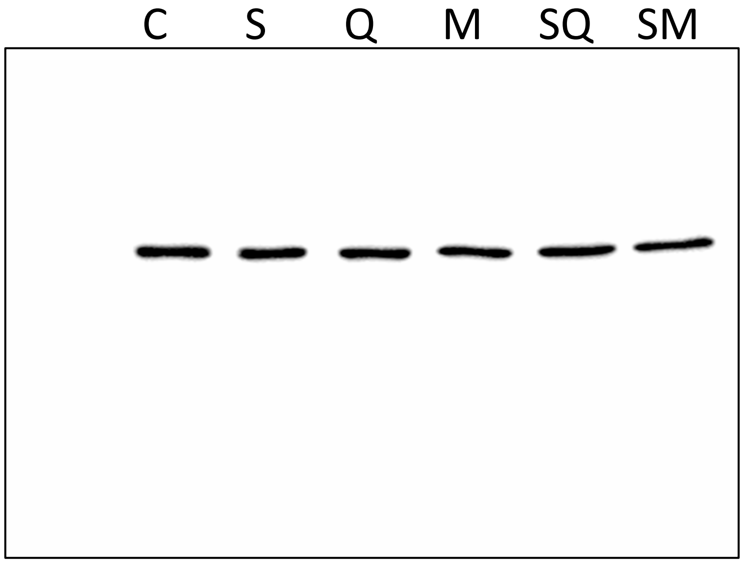

NDP52 – 52 kDa (merged)

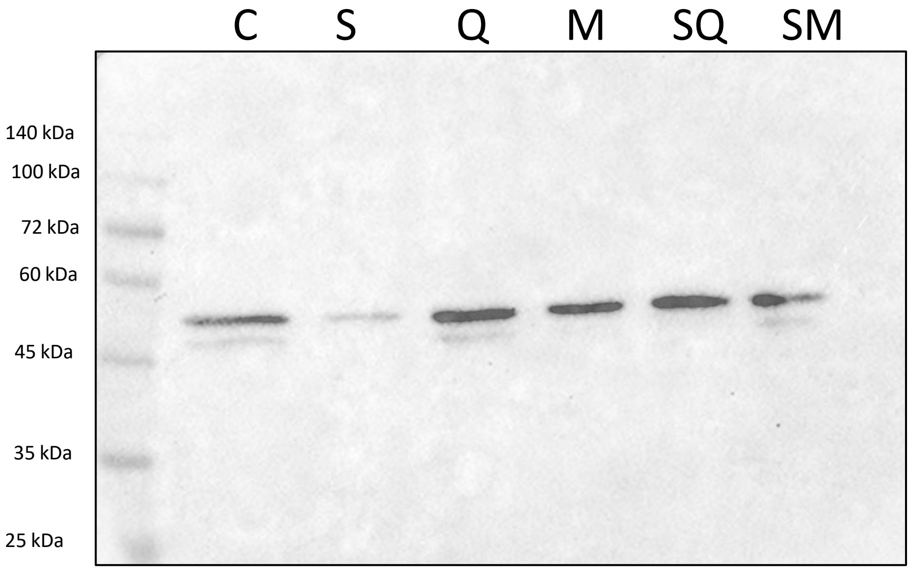

β -Actin -42kDa (merged)

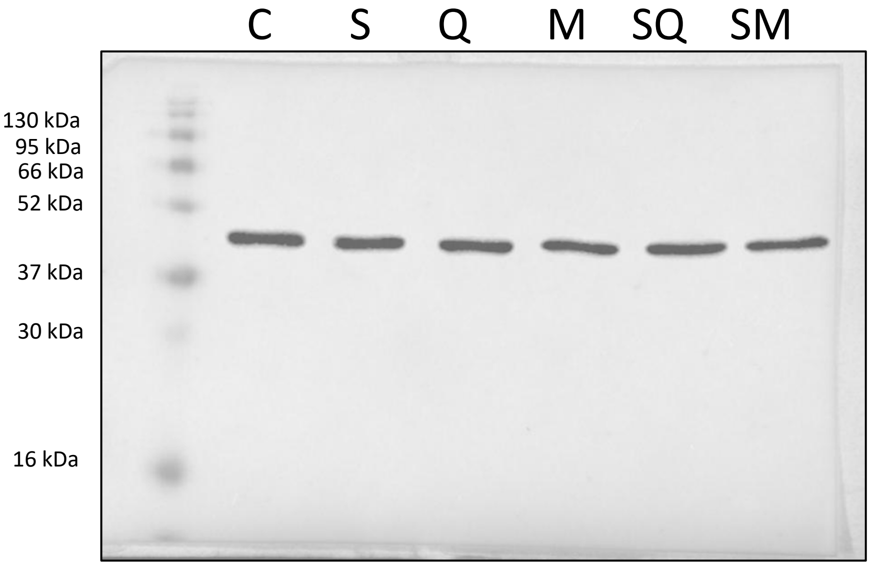

BNIP3- 22-28kDA , 50-55kDa (Chemi Doc)

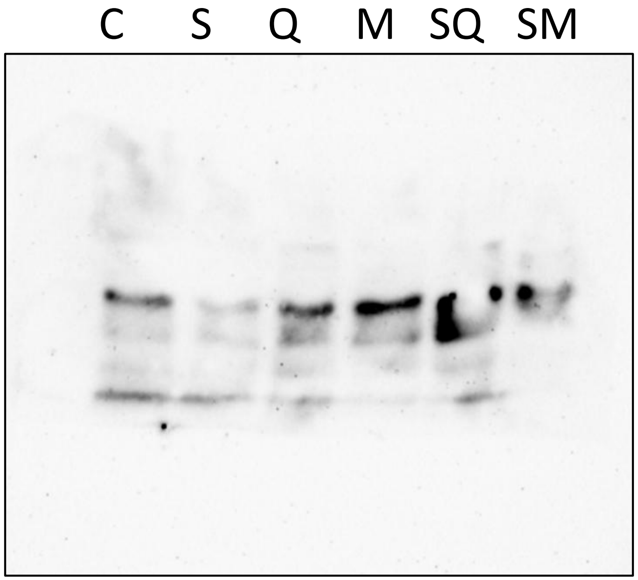

β-Actin -42kDa (Chemi Doc)

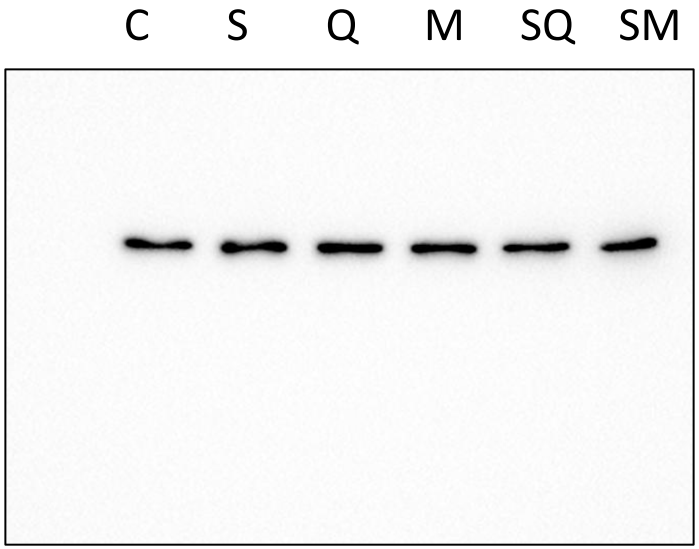

BNIP3- 22-28kDA , 50-55kDa (merged)

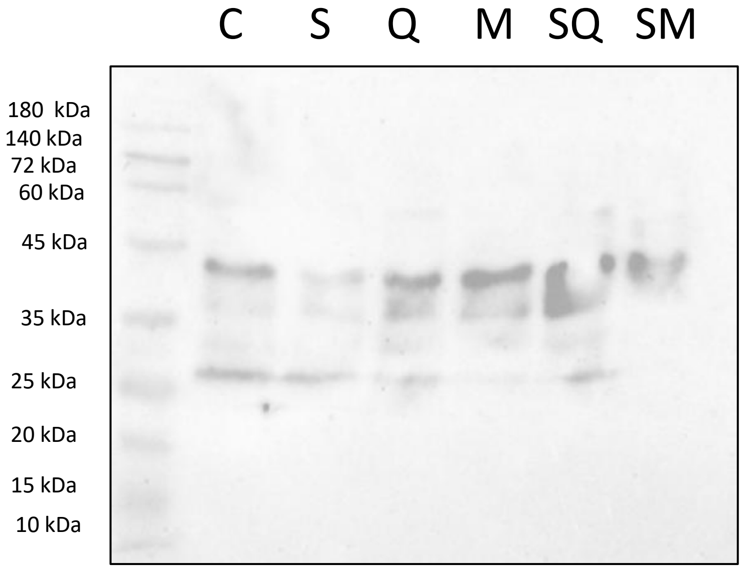

β-Actin -42kDa (merged)

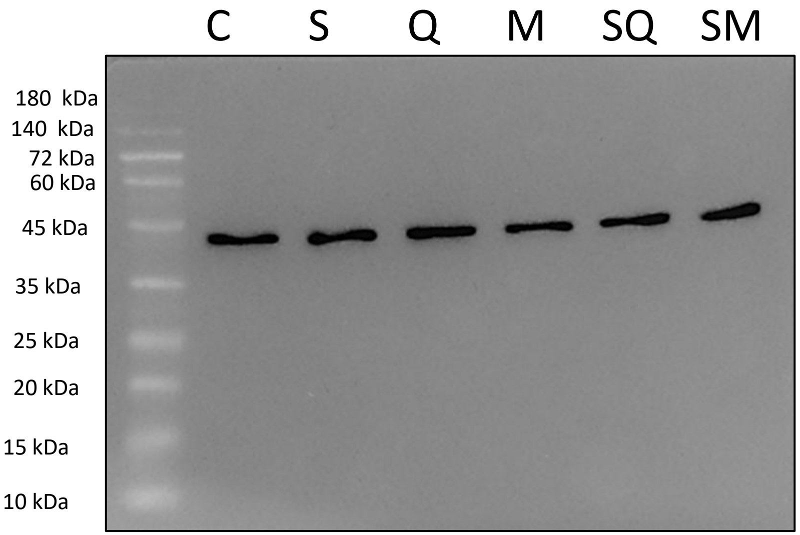

BNIP3L/Nix – 38kDa (Chemi Doc)

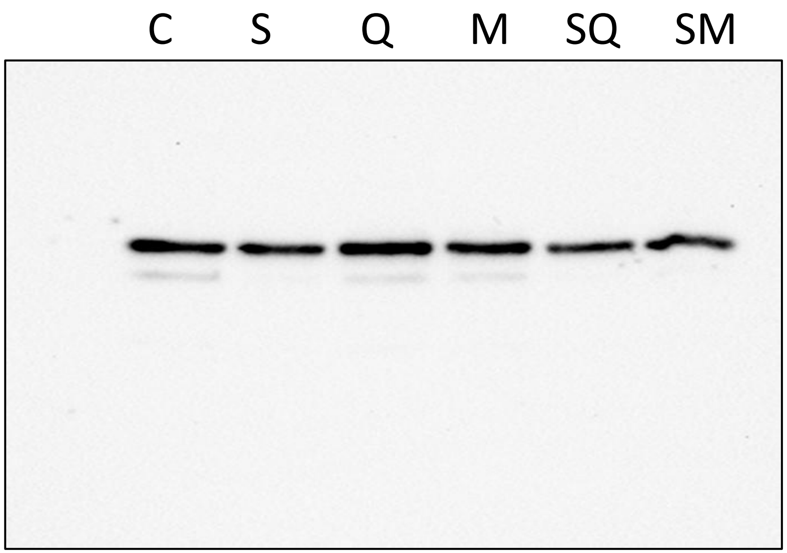

β -Actin -42kDa (Chemi Doc)

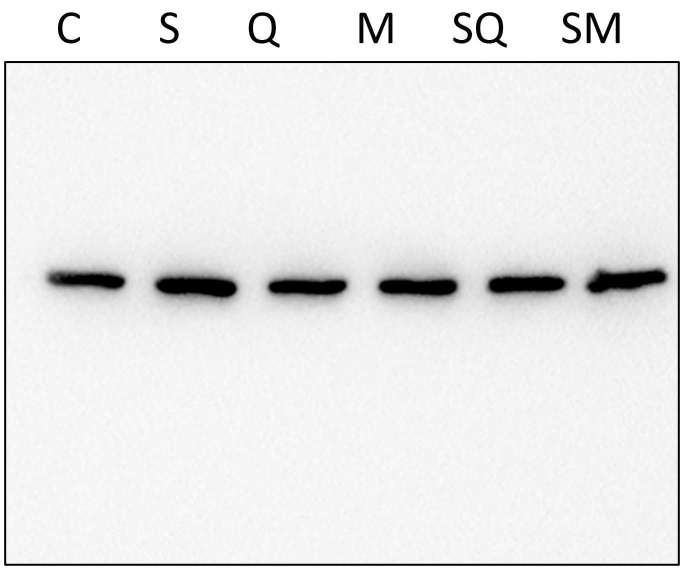

BNIP3L/Nix- 38 kDa (merged)

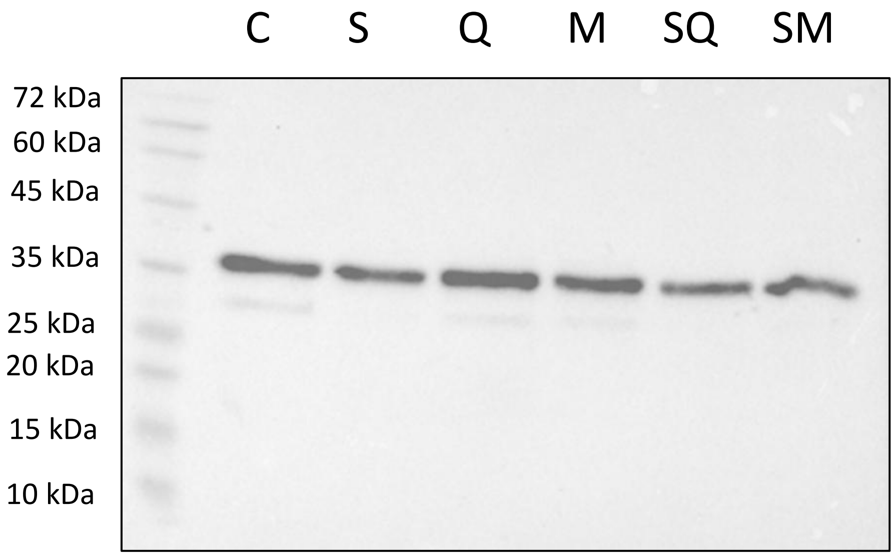

β -Actin -42kDa (merged)

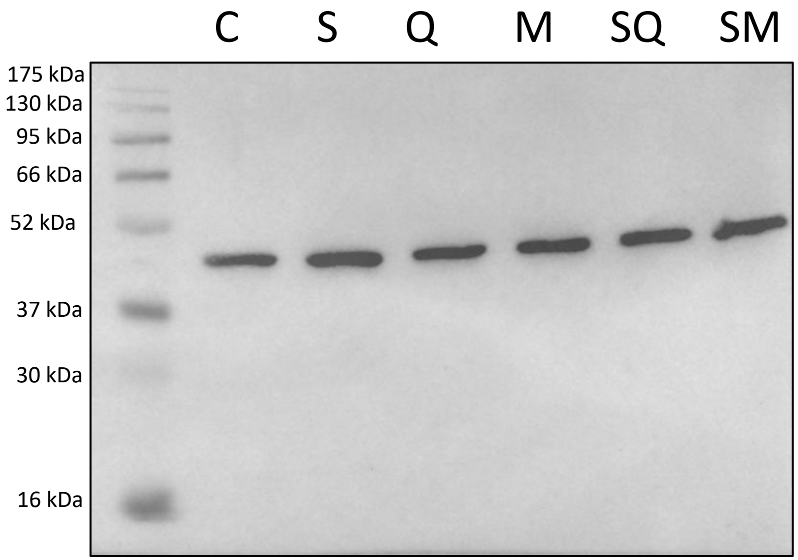

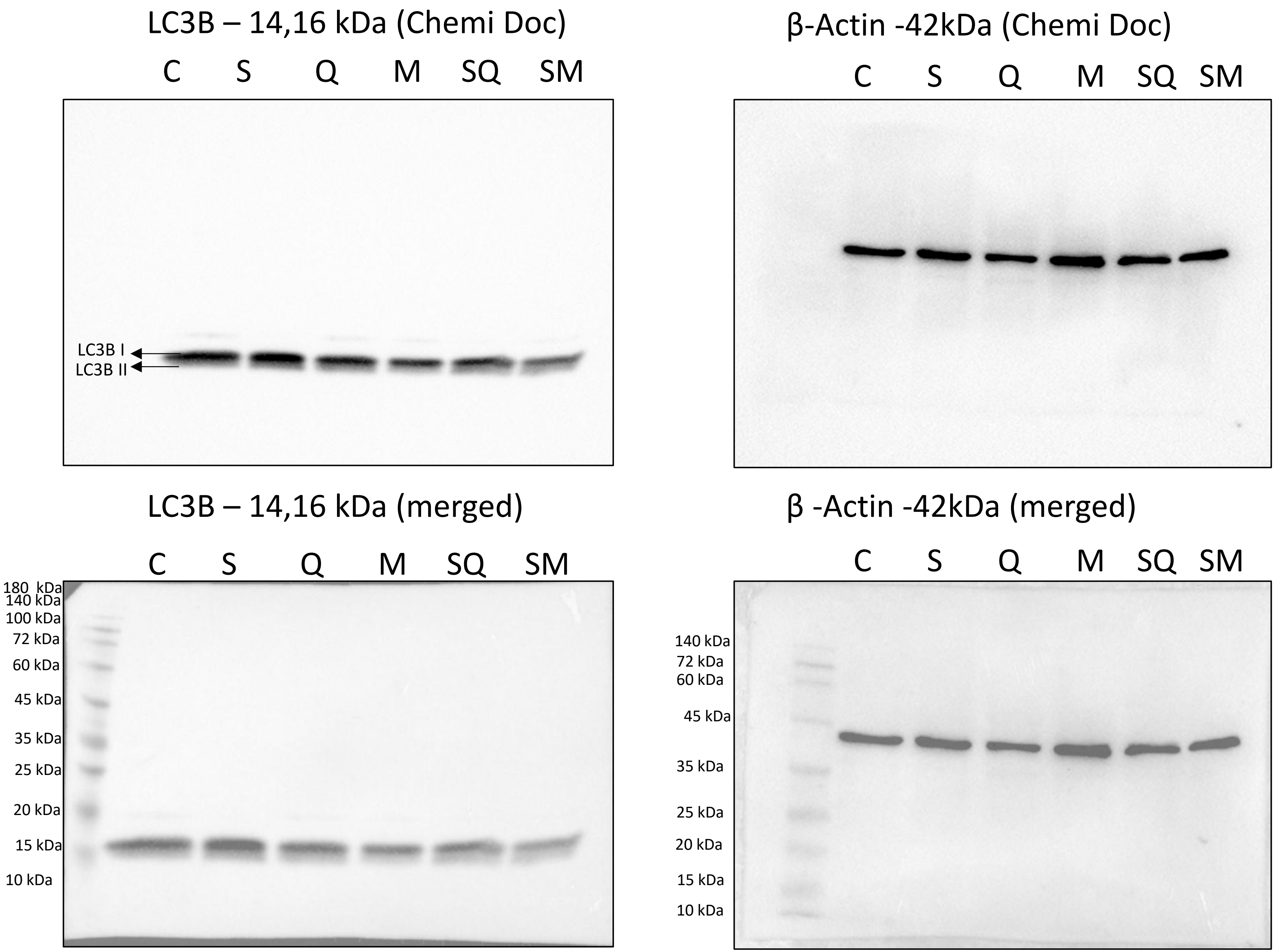
LC3B – 14,16 kDa (Chemi Doc)
C S Q M SQ SM
LC3B I
LC3B II
β-Actin -42kDa (Chemi Doc)
C S Q M SQ SM
LC3B – 14,16 kDa (merged)
C S Q M SQ SM
180 kDa
140 kDa
100 kDa
72 kDa
60 kDa
45 kDa
35 kDa
25 kDa
20 kDa
15 kDa
10 kDa
β -Actin -42kDa (merged)
C S Q M SQ SM
140 kDa
72 kDa
60 kDa
45 kDa
35 kDa
25 kDa
20 kDa
15 kDa
10 kDa
