# Supplementary figures and images for: Melatonin and Coenzyme Q10 mitigate Senescence in Human Adipose-Derived Mesenchymal Stem Cells by Restoring Mitophagy and Mitochondrial Proteostasis
Source: PLoS One. 2026 Apr 29;21(4):e0347781. doi: 10.1371/journal.pone.0347781 (PMC13128124; doi:10.1371/journal.pone.0347781)

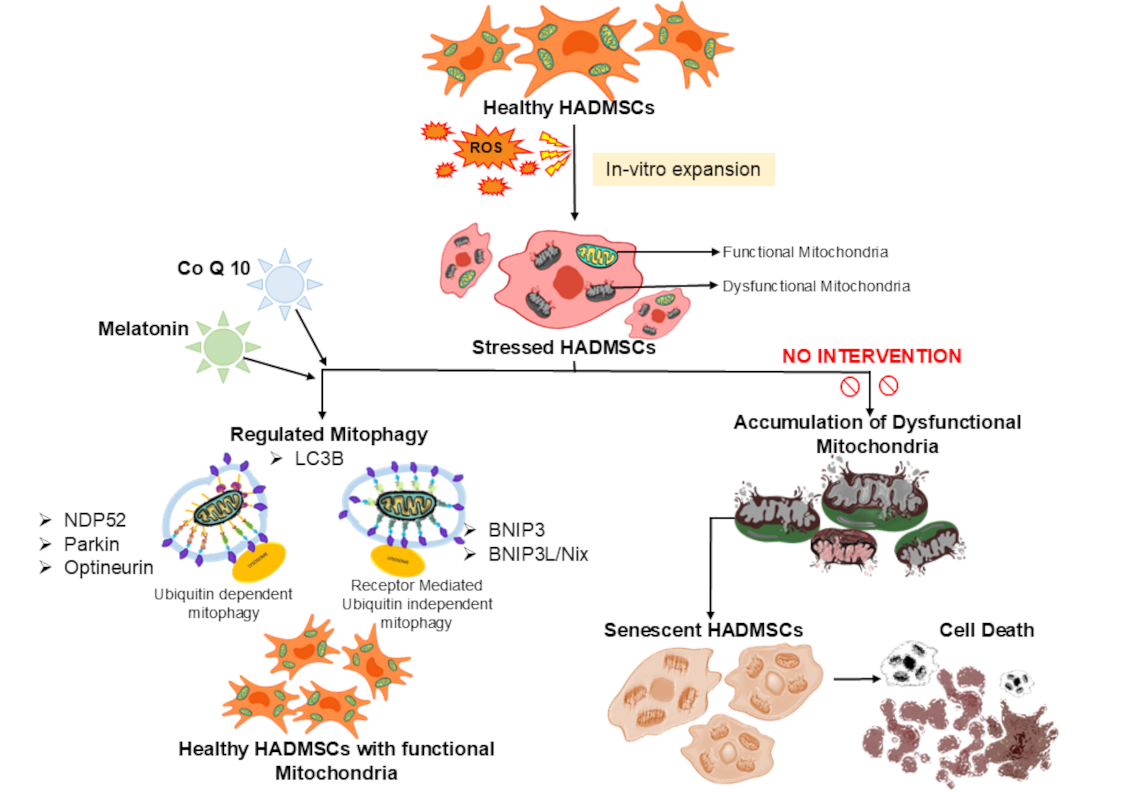

Supplement: S2 Fig — (TIF) [file pone.0347781.s002.tif]
