# Supplementary material for: Melatonin and Coenzyme Q10 mitigate Senescence in Human Adipose-Derived Mesenchymal Stem Cells by Restoring Mitophagy and Mitochondrial Proteostasis
Source: PLoS One. 2026 Apr 29;21(4):e0347781. doi: 10.1371/journal.pone.0347781 (PMC13128124; doi:10.1371/journal.pone.0347781)
Supplement: Attachment — Submitted filename: Response to Reviewers.docx [file pone.0347781.s004.docx]

**Response to Reviewers**

**Manuscript ID: PONE-D-25-60616R2**

We sincerely appreciate the detailed feedback of the reviewers which had helped us to improvise our manuscript in this second revision. We have carried out the corrections as mentioned by the reviewer and journal requirements. We had re-viewed our references cited in our manuscript to ensure that none of the citations are made from retracted papers. We have cross-checked every reference with Google Scholar and Zotero to identify retracted status of any of the citations. There are no changes in the reference list. We have provided a point-by-point response to reviewer’s comments.

Reviewer #1:

While the revised manuscript has improved scientifically, two major issues remain.

Comment: 1. Title

The current title overstates the scope of the findings. Terms such as “synergistic role” and “metabolic reprogramming” imply mechanistic conclusions that are not directly supported by the presented data. The study demonstrates antioxidant-mediated protection and modulation of mitophagy-related protein expression, but does not provide direct evidence of mitophagy flux or metabolic reprogramming. The title should therefore be revised to more accurately reflect the experimental evidence.

Author’s Response:

In accordance to your suggestions and acknowledging the limitations of the presented data, we have changed the title and it is now titled as **“Melatonin and Coenzyme Q10 mitigate Senescence in Human Adipose-Derived Mesenchymal Stem Cells by Restoring Mitophagy and Mitochondrial Proteostasis”.**

Comment: 2. Language and Clarity

The manuscript still requires substantial language revision. There are multiple grammatical inconsistencies and unclear constructions throughout the text. For example:

- Missing conjunctions (e.g., “differentiate into multiple lineages, improve metabolic homeostasis”).
- Incorrect or unnecessary verb usage (e.g., “Premature senescence in MSCs is caused…”).
- Repeated sentence (e.g., "117% and 98.8%")
- Inconsistent formatting of concentrations (e.g., 400Nm).
- Abbreviations such as “Str” or “STR” not clearly defined at first mention.
- P-values placed mid-sentence rather than at the end of statements.

A thorough professional language editing and careful proofreading are strongly recommended.

Author’s Response:

Thank you for your detailed feedback regarding the language and clarity of manuscript. The examples mentioned, have been corrected in our manuscript. This helped us to fix the missing conjunctions and overall grammatical aspect of all the sentences.

- Missing conjunctions: (e.g., “differentiate into multiple lineages, improve metabolic homeostasis”- fixed in Page 2, line 23-25.
- Incorrect verb usage: (e.g., “Premature senescence in MSCs is caused…”- fixed in Page 3, line 3-5.
- Repeated sentence: 117% and 98.8%"- removed
- Inconsistent formatting of concentrations (e.g., 400Nm) has been corrected in Page 8, line 23.
- Abbreviations such as “Str” or “STR” has been corrected to “STR” throughout the manuscript and STR has been clearly mentioned as Stressed (STR) at first mention in page 10 and line 27.
- P-values initially placed mid-sentence rather than at the end of statements has been corrected and placed at the end of the statements, throughout the manuscript as per the journal style.

We have conducted line by line proof-reading to eliminate grammatical errors, confusing sentences or any inconsistencies like mentioned in the comments.

Reviewer #2: In this manuscript, authors showed that antioxidants (melatonin and CoQ10) restore mitophagy in stressed HADMSCs by reducing ROS and lipid peroxidation, enhancing mitophagy markers (Parkin, NDP52, BNIP3, Nix, LC3B), and reversing cellular senescence. This improves stem cell survival, metabolic function, and therapeutic potential for transplantation. The authors have addressed all my comments. I recommend to accept this manuscript.

Author’s Response:

Thank you for your time and effort for reviewing our manuscript. Your comments provided during the first revision has helped us immensely improve the quality of our manuscript. We are grateful for your insightful suggestions and thorough evaluation. **Our heartfelt thanks for your positive assessment and your recommendation for the acceptance of our manuscript.**
